# Effects of dimethyloxalylglycine on wound healing of palatal mucosa in a rat model

**DOI:** 10.1186/s12903-015-0047-1

**Published:** 2015-05-16

**Authors:** Tingting Zhu, Hee Chul Park, Kyung Mi Son, Hyeong-Cheol Yang

**Affiliations:** Department of Dental Biomaterials Science and Dental Research Institute, College of Dentistry, Seoul National University, 28 Yeonkun-dong, Chongro-ku, Seoul 110-749 South Korea

**Keywords:** Dimethyloxalylglycine, Hypoxia-inducible factor 1 alpha, Vascular endothelial growth factor, Palatal mucosa, Wound healing

## Abstract

**Background:**

Rapid wound healing of oral soft tissue may reduce the opportunity of infection and discomfort of patients. Previous studies have demonstrated that enhancement of angiogenesis is an effective way to accelerate wound repair. In this study, to enhance angiogenesis and healing of palatal wounds, dimethyloxalylglycine (DMOG) was applied to a rat palatal wound model. DMOG is known to inhibit oxygen-dependent degradation of hypoxia inducible factor-1 alpha (HIF-1α), which can lead to up-regulation of angiogenesis markers, favoring wound repair. We also evaluated the effects of DMOG on cell migration and HIF-1α expression of rat palatal (RP) cells. Furthermore, mRNA and protein expression of vascular endothelial growth factor (VEGF) were analyzed in DMOG-treated RP cells.

**Methods:**

Primary cultures of rat palatal (RP) cells were obtained from Sprague–Dawley (SD) rats. Effects of DMOG on cell viability and migration of RP cells were evaluated by using a formazan and culture insert, respectively. VEGF mRNA was observed by real-time PCR, and VEGF and HIF-1α proteins were detected by Western blotting. For the animal study, excisional wounds, 3 mm in diameter, were made at the central part of the palate of SD rats. DMOG with hyaluronic acid ointment was topically applied three times during 1 week, and then wound closures were quantitated photographically and histologically.

**Results:**

DMOG was cytotoxic to RP cells at concentrations higher than 2 mM and did not affect cell migration at non-cytotoxic concentrations. mRNA and protein expression of VEGF were significantly stimulated by DMOG treatment. The protein level of HIF-1α was also stabilized in RP cells by DMOG. In the animal study, groups treated with 1 mg/ml DMOG showed an increase of rat palatal wound contractures.

**Conclusions:**

DMOG enhanced wound healing of rat palatal mucosa, which was likely due to the angiogenic effect of the agent.

## Background

Oral soft tissues are inevitably damaged during autogenous gingival grafting, periodontal surgery, or dental implant surgery. Recovery of soft tissue from injuries depends on wound size, location, condition of oral hygiene, and the level of immunity. Oral administration or topical application of antibiotics is recommended as a choice of oral wound care to prevent bacterial infection. In the case of large-sized defects that can be caused by autogenous gingival grafts, dressing materials are used to protect damaged donor sites and to help the wound healing process. Intended rapid wound healing can also reduce the opportunity of infection and discomfort of patients. In a previous study, acceleration of palatal mucosa repair was achieved with a collagen-gelatin scaffold retaining basic fibroblast growth factor (bFGF), a potent angiogenesis inducer [1]. Thymosin β_4_ (Tβ_4_), an oligopeptide that can sequester G-actin monomers, also promotes wound healing of rat mucosa [2]. Enhancement of cell migration and angiogenesis is likely to be involved in wound healing by Tβ_4_. Umeki et al. have shown that wound healing of oral mucosa is accelerated by leptin, a circulating anti-obesity hormone, via enhancement of angiogenesis [3]. These studies imply that enhancement of angiogenesis is an effective way to accelerate oral wound repair.

Angiogenesis can be stimulated by various growth factors such as fibroblast growth factor (FGF) [4], vascular endothelial growth factor (VEFG) [5], platelet-derived growth factor (PDGF) [6, 7], and transforming growth factor β (TGF-β) [8, 9]. Several peptides have also been reported to promote angiogenesis [10–12]. Considering the importance of angiogenesis in wound healing, the application of angiogenic proteins and peptides to wounds is expected to expedite the repair of oral tissue damage if the molecules are properly delivered to the wound sites. However, the stability and activity of proteins and peptides cannot be assured in the oral environment where temperature and pH are changeable due to eating and drinking. Instead, angiogenic small molecules which are chemically stable can be more appropriate for the harsh conditions of the oral cavity.

Dimethyloxalylglycine (DMOG), with small molecular weight, is a cell-permeable unspecific inhibitor of prolyl hydroxylases (PHDs) [13]. Under normoxic conditions, PHD2 is known to hydroxylate specific proline residues in HIF-1α, which then leads to degradation of HIF-1α by the ubiquitin-proteasome pathway [14–16]. Therefore, inhibition of PHD2 by DMOG can prevent HIF-1α degradation, creating an environment similar to that found in hypoxic cells. Furthermore, various genes related to tissue repair such as angiogenesis are upregulated. A previous study showed that DMOG enhanced the production of VEGF in periodontal fibroblast cells [17]. Furthermore, Botusan et al. demonstrated that DMOG stabilizes HIF-1α and enhances dermal wound healing in diabetic mice [18]. In this study, the effect of DMOG on oral wound healing was investigated in a rat palatal wound model. We also evaluated the effects of DMOG on cell migration and HIF-1α expression of rat palatal (RP) cells. Furthermore, mRNA and protein expression of vascular endothelial growth factor (VEGF) were analyzed in DMOG-treated RP cells.

## Methods

### Chemical reagents and cell culture

Cell culture medium and antibiotics were purchased from WELGENE Inc. (Daegu, Korea). Fetal bovine serum (FBS) was purchased from Lonza (Basel, Switzerland), and other experimental reagents were obtained from Sigma-Aldrich Co. (Saint Louis, MO, USA), unless otherwise specified.

RP cells were isolated from the palatal tissues of 5-week old male Sprague–Dawley (SD) rats. Isolated palatal tissues were washed with phosphate buffered saline (PBS) (PH 7.4), aseptically minced into pieces, and then immersed in Dulbecco’s modified Eagle’s medium (DMEM) containing 10% FBS and antibiotic solution (100 U/ml of penicillin-G and 100 μg/ml of streptomycin) at 37 °C in a humidified incubator (5% CO_2_/95% air). After 20 days of culture with medium changes at 3-day intervals, RP cells were collected and sub-cultured under the same conditions. Passages three through five were used for this study. Although we did not identify the type of palatal cells at the molecular level, morphological features of the isolated cells under the microscope indicated distinct dominance of fibroblast-like cells.

### Cytotoxicity

RP cells incubated until 80% confluent were removed and sub-cultured at 0.8 × 10^5^ cells per ml in 96-well plates and incubated at 37 °C with 5% CO_2_ in air for 24 h, and then the cells were treated with DMOG at various concentrations ranging from 0.1 to 10 mM. After treatment for 24 h, the cell viabilities were quantified using the Cell Counting Kit-8 (WST-8) (Dojindo Laboratories, Kumamoto, Japan). Cells were incubated in 100 μl of WST-8 solution for 1 h at 37 °C in a humidified atmosphere (5% CO_2_/95% air). The absorbance was measured at a wavelength of 450 nm using a plate reader (Sunrise, TECAN, Salzburg, Austria). The optical density of untreated cells was designated as 100%, and cell viability of treated cells was expressed as the percentage of untreated negative control.

### Cell migration assay

For the *in vitro* cell migration assay, a culture insert (ibidi GmbH, Martinsried, Germany) was used to create a wound in cell culture. The culture insert was placed on a culture dish, and 70 μl of RP cell suspension (5 × 10^5^ cells/ml) was added into both wells of the insert. The RP cells were incubated at 37 °C for 48 h and then exposed to DMOG (0, 0.1, 0.5, 1, 2 mM) in culture media containing 2% FBS for the cell migration analysis. Wound closure was observed and recorded at intervals under a phase contrast microscope (Olympus, Tokyo, Japan). To quantify cell migration, the uncovered area where no cells were present was measured by using ImageJ program, and the ratio of uncovered area between untreated control and treated groups was obtained.

### mRNA expression analysis by real-time PCR

The effect of DMOG on the expression of VEGF mRNA was investigated by real-time polymerase chain reaction (RT-PCR) assay. After treatment with DMOG at 0, 0.1, 0.5, 1, and 2 mM for 24 h, total RNA was isolated using RNA extraction reagent (WelPrep Total RNA Isolation Reagent, WELGENE Inc.). From the total RNA, cDNA was prepared using a cDNA synthesis kit (Power cDNA Synthesis Kit, iNtRON Biotechnology, Sungnam, Korea), and RT-PCR was performed in an ABI PRISM 7500 Sequence Detection System Thermal Cycler (Applied Biosystems, Foster City, CA, USA) with 20 μl reaction volumes containing 10 μl SYBR premix Ex Taq (Takara Bio, Otsu, Japan), 0.4 μl ROX Reference Dye II (Takara Bio), cDNA, and primers. The primers for gene amplification were as follows: VEGF sense, 5’-GAGTATATCTTCAAGCCGTCCTGT-3’; VEGF antisense, 5’-ATCTGCATAGTGACGTTGCTCTC-3’; GAPDH (Glyceraldehyde-3-phosphate dehydrogenase) sense, 5’-TGTGTCCGTCGTGGATCTGA-3’; GAPDH antisense, 5’-CCTGCTTCACCACCTTCTTGAT-3’. The PCR conditions were 95 °C for 30 s, followed by 40 cycles of denaturation at 95 °C for 5 s and annealing at 63 °C (34 s) for VEGF. All reactions were run in triplicate. Gene expression was evaluated on the basis of the threshold cycle (CT value) and normalized to the expression of the GAPDH gene.

### Western blot analysis

Western blot analysis was performed to examine the protein expression of HIF-1α and VEGF in DMOG-treated palatal cells. After treatment with DMOG at various concentrations for 24 h, cells were lysed in extraction buffer containing 50 mM Tris base-HCl (PH 7.5), 150 mM NaCl, 0.5% Triton-X 100, and one tablet of protease inhibitor cocktail (1 tablet/10 ml, Roche Applied Science, Mannheim, Germany) for 45 min on ice. Extracts containing equal amounts of protein were run on 10% sodium dodecyl sulfate polyacrylamide gels and transferred to polyvinylidene difluoride membranes. The blots were incubated with rabbit polyclonal antibodies against VEGF, HIF-1α, or GAPDH in PBST (PBS containing 0.1% Tween 20) for 1.5 h, washed three times with PBST, and then probed with goat anti-rabbit secondary antibodies conjugated to horseradish peroxidase. The protein bands were visualized using a chemiluminescence kit (WEST-ZOL plus Western Blot Detection System, iNtRON Biotechnology). Chemiluminescence was detected using the LAS 1000 Plus Luminescent Image Analyzer (Fuji Photo Film, Tokyo, Japan). All antibodies were purchased from Santa Cruz Biotechnology (Santa Cruz, CA, USA).

### Rat palatal wound healing assay

After confirming the angiogenesis effect of DMOG on RP cells, the effect of DMOG on wound healing of palatal tissue was performed in a rat palatal wound model. All rats were housed under specific pathogen-free (SPF) conditions at the animal experimental center of Seoul National University Dental School. Eighteen 13-week-old male SD rats (six rats for each group), weighing 300–350 g, were used in the present study. All animal experiments were approved by the Institutional Animal Care and Use Committee (SNU-130123-8-1) of Seoul National University (Seoul, Korea). Under general anesthesia, punch wounds were made on a central area of hard palate with a disposable 3-mm diameter biopsy punch (Kai Industries Ltd., Gifu, Japan), exposing a circular area of bare bone. Hyaluronic acid (HA) ointment (20 mg/ml) containing 0, 0.5, or 1 mg/ml (5.7 mM) DMOG was applied to the wound area. After the surgery, animals were fed a standard diet of pellets and water with enrofloxacin. The agents were re-applied on days 2 and 4 under anesthesia to reduce stress, and the rats were sacrificed on day 7. The hard palate was separated, and the wound area was observed with a stereoscopic microscope (Nikon, Tokyo, Japan) and by histological analysis. The wound area on the microscopic images was calculated using CellSense Dimension 1.6 software (Olympus, Tokyo, Japan). Palatal specimens were taken for histomorphometric evaluation and samples were stained with haematoxylin and eosin (H&E) staining. More than ten slides for each sample were prepared, and we selected a section that exhibited the widest diameter of wound for histological analysis. The sections were examined under a light microscope (Olympus), and the distance of wound margins in each section was measured with a calibrated ocular micrometer.

### Statistical analysis

For statistical analysis, each experiment was performed in triplicate unless otherwise specified. The data are expressed as the mean ± Standard Deviation. Statistical analyses were determined by one-way ANOVA for in vivo study and cell migration assay. The unpaired Student’s *t*-test was used for the other *in vitro* results. A *P*-value < 0.05 was considered statistically significant.

## Results

### Cell viability and migration of DMOG-treated rat palatal cells

**Fig 1:**
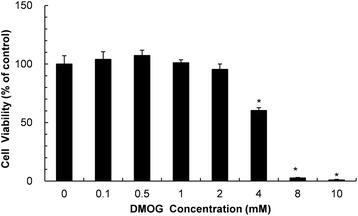
Effect of DMOG on the viability of RP cells. RP cells were treated with DMOG at concentrations ranging from 0 to 10 mM for 24 h. Data and error bars indicate means ± SD of three independent experiments. *demonstrates a statistically significant (*P* < 0.05) difference between control and treated cells

**Fig 2:**
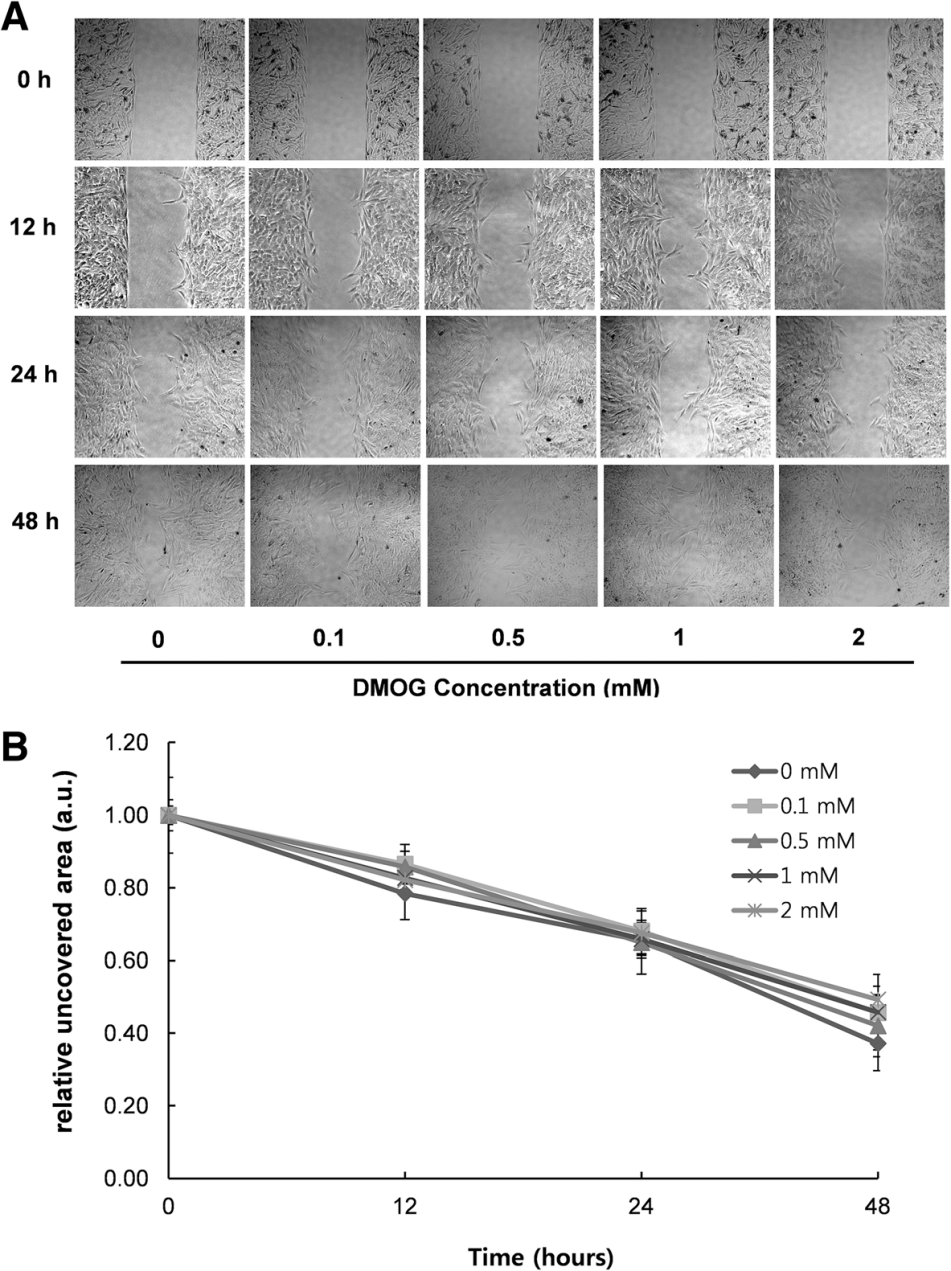
Cell migration of DMOG-treated RP cells. (**A**) Representative photomicrographs of a cell migration assay. RP cells were treated with DMOG at different concentrations in culture media containing 2% FBS for the cell migration assay with 40X magnification. (**B**) Quantitative analysis of cell migration. Cell migration was quantified by calculating the ratio between a cell-free and cell-occupied area inside the wound space. No statistically significant differences appeared among all groups at the same time point (*P* < 0.05)

To investigate the effects of DMOG on cell viability, rat palatal cells were exposed to DMOG for 24 h. The cytotoxicity of DMOG was demonstrated at concentrations higher than 2 mM. Cell viability was reduced to 60% at 4 mM, and was mostly impaired at 8 mM (Fig. 1). Cell migration was observed in culture media containing 2% FBS to attenuate cell motility. In the absence of FBS, untreated palatal cells did not display any movement during 48 h (data not shown). In 2% FBS medium, a slight movement of cells was shown at 12 h, and about a half of the void area was filled with penetrated cells at 48 h. Cell migration during 48 h was not affected by DMOG at non-cytotoxic concentrations (0–2 mM) (Fig. 2).

### mRNA and protein expression of the VEGF gene and HIF-1α protein levels in DMOG-treated cells

**Fig 3:**
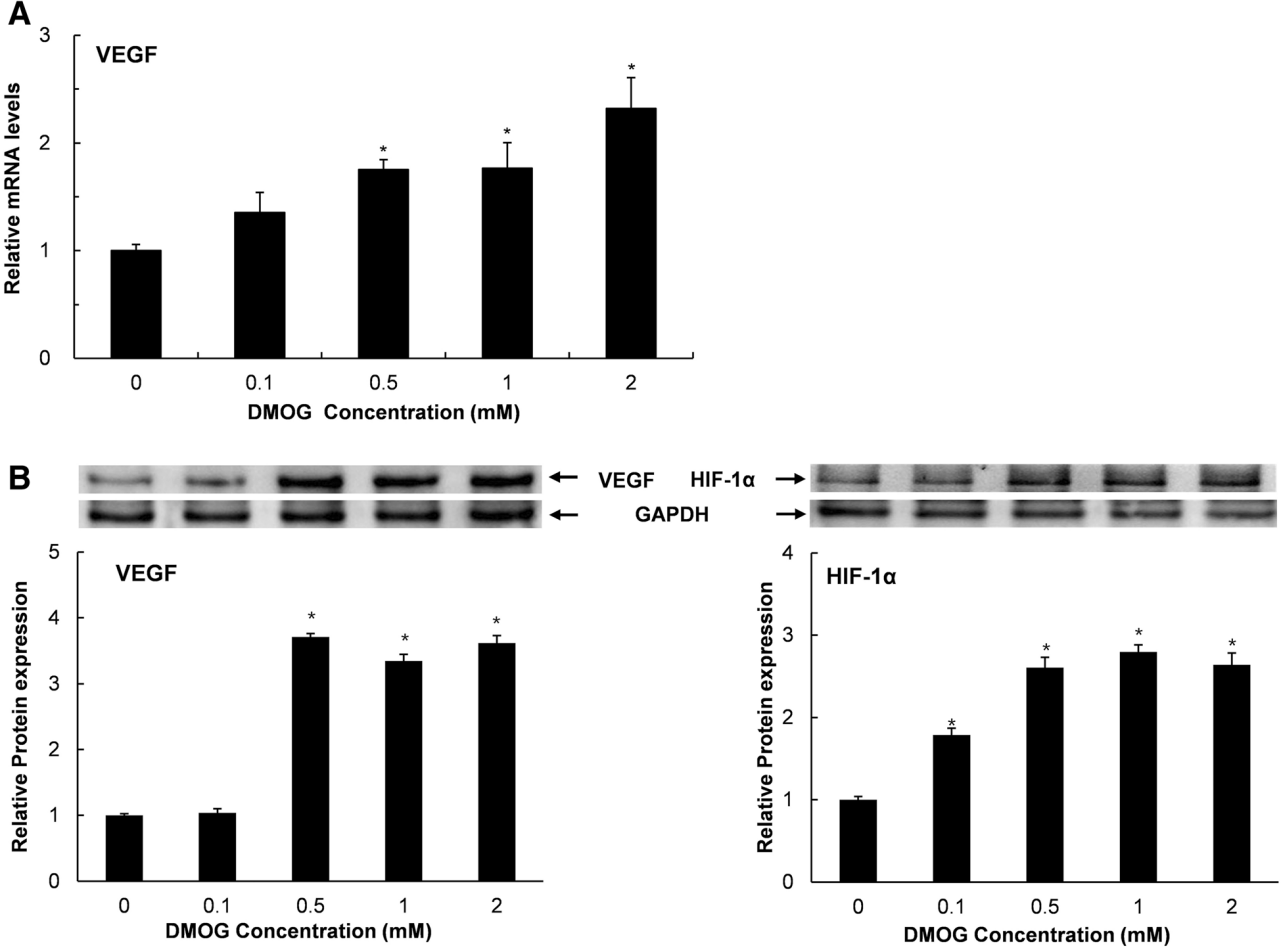
mRNA expression of the VEGF gene and protein expression of VEGF and HIF-1α in DMOG-treated cells. RP cells were treated with DMOG at concentrations ranging from 0 to 2 mM for 24 h. Data and error bars indicate means ± SD of three independent experiments. *demonstrates mRNA (**A**) and protein (**B**) expression levels that are significantly different from those of untreated control cells (*P* < 0.05)

mRNA and protein expression of VEGF was quantified to investigate the effects of DMOG on angiogenesis at the molecular level. As shown in Fig. 3A, DMOG enhanced gene expression of VEGF dose-dependently in RP cells. Treatment with DMOG at 0.5 mM showed an increase in the amount of VEGF mRNA that was significantly higher than that of the control group, and 2 mM DMOG caused a 2.3-fold increase in mRNA levels (Fig. 3A). The protein expression of VEGF was also up-regulated by DMOG at concentrations higher than 0.5 mM. Unlike the dose-dependent pattern of mRNA expression in DMOG-treated cells, a 3.4-fold higher level of protein was maintained at DMOG concentrations between 0.5 and 2 mM (Fig. 3B).

The stability of HIF-1α protein in RP cells was also affected by DMOG. Greater amounts of HIF-1α protein were detected in cells treated with DMOG even at 0.1 mM which did not cause a change in the protein level of VEGF (Fig. 3B). A 2.6-fold increase in HIF-1α protein levels was shown in cells that were treated with 0.5 mM DMOG. No further significant increase in protein levels was shown at higher concentrations, indicating that 0.5 mM of DMOG is enough to stabilize the entire amount of HIF-1α protein in cells.

### The effects of DMOG on palatal wound healing in rats

**Fig 4:**
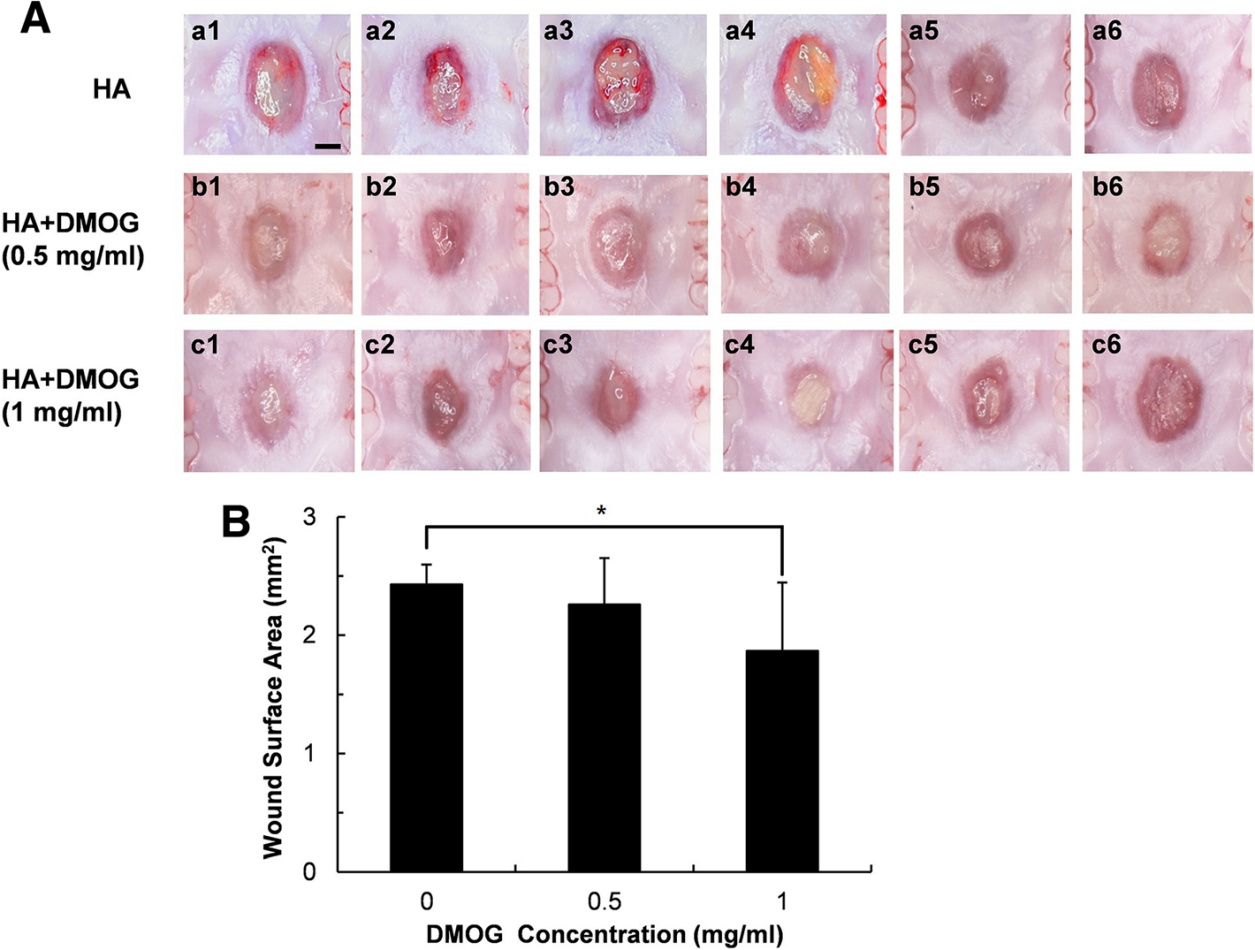
Effects of DMOG on healing of palatal wounds. (**A**) Stereoscopic microscope images of palatal wounds 1 week after surgery. DMOG was topically applied to test groups (*b1*–*b6*: 0.5 mg/ml, *c1*–*c6*: 1 mg/ml), and vehicle (20 mg/ml HA) was applied to control groups (*a1*–*a6*) (Scale bar: 1 mm). (**B**) Wound area calculated from the stereoscopic microscope images. *indicates a significant difference in the palatal wound area compared to that of the untreated control groups (*P* <0.05)

In a rat palatal wound model, DMOG was applied at two concentrations: 0.5 and 1 mg/ml. The DMOG-treated group did not show any symptoms of physiological abnormality during the experimental period. As shown in Fig. 4A, the wound area was not completely epithelized at 1 week, and was readily distinguished from undamaged intact tissue that was pale pink in color. The average area of the wound surface in the control group was 2.4 mm^2^. In the test group treated with DMOG at 1 mg/ml, the wound area was reduced to 1.8 mm^2^, which is modest but still a statistically significant decrease. At 0.5 mg/ml, DMOG did not affect wound closure.

**Fig 5:**
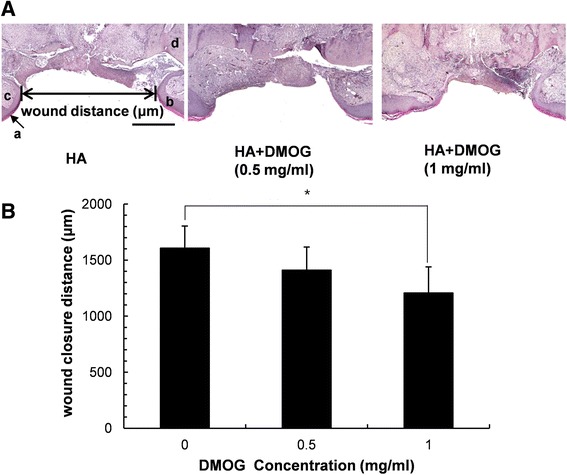
Histological observation. (**A**) Representative haematoxylin and eosin (H&E) staining photographs (*a*: stratum corneum, *b*: epithelium, *c*: connective tissue, *d*: maxillae) (Scale bar: 500 μm). (**B**) Mean values of maximum palatal wound width calculated from the HE-stained sections. *indicates a significant difference in palatal wound width of DMOG-treated groups compared to that of the untreated control groups (*P* < 0.05)

To confirm the results shown in Fig. 4A, the maximum distance of the unepithelized region (distance of wound margin) was measured in histological sections of the wound area. The distance was also reduced in rats that were treated with 1 mg/ml DMOG, while there was no statistically significant difference in the distance of the wound margin between control and 0.5 mg/ml DMOG-treated groups (Fig. 5). Therefore, DMOG clearly accelerated palatal wound healing in rats at a certain concentration.

## Discussion

HIF prolyl hydroxylase inhibitors have been investigated to find a new drug for treatment of anemia, kidney disease, and heart failure [19, 20]. Promotion of angiogenesis is a targeted therapeutic effect of the newly developing drugs, because angiogenesis is an inevitably necessary step in tissue regeneration or wound healing.

We observed the effect of DMOG, a HIF-1α prolyl hydroxylase inhibitor, on wound healing of rat palatal mucosa. For *in vitro* studies, cytotoxicity and the ability to regulate VEGF and HIF-1α in cells originating from rat palatal tissues were investigated. The cytotoxicity of DMOG against rat palatal cells appeared at relatively higher concentrations (Fig. 1). Accumulation of HIF-1α protein is not likely a direct cause of cell death because the intracellular level of HIF-1α was already saturated at 0.5 mM and was not altered until 2.0 mM. A previous study reported cell cycle arrest by PHD inhibitors [21], which provides a possible explanation for the toxic effects of DMOG. Since cytotoxicity undoubtedly can disturb tissue repair, DMOG concentrations for in vivo studies should be carefully chosen to elicit a wound healing effect. Marchbank et al. demonstrated that DMOG stimulates migration of human stomach and colonic carcinoma cells [22]. However, a decrease in single cell migration has been observed in the hypoxia state of certain fibroblast cells [23]. Therefore, the effect of hypoxia or HIF-1α accumulation on cell migration is cell-type specific. The motility of rat palatal cells in this study was not promoted by DMOG (Fig. 2), which suggests that migration of fibroblast-like palatal cells was not a determining factor in wound healing of DMOG-treated palatal cells.

Previously, it has been demonstrated that PHD inhibitors up-regulate the expression of VEGF in various kinds of cells, including endothelial cells [24], epithelial cells [25], gingival fibroblasts, and periodontal ligaments [17]. In agreement with the results of these studies, the present study also confirmed the ability of DMOG to up-regulate the expression of VEGF (Fig. 3) in rat palatal fibroblast-like cells. DMOG also induced stabilization of HIF-1α protein, and the range of effective concentrations was overlapped except for 0.1 mM at which only HIF-1α protein but not VEGF was up-regulated. This indicates that VEGF cannot be induced in the absence of obvious increases in the amount of HIF-1α. Additionally, the stabilization of HIF-1α is also known to induce glucose transporter-1 and phosphoglycerate kinase-1, which can improve the wound healing by enhancing the re-epithelialization [26].

Because of the moist condition of oral cavity in the animal experiment, it was required to employ a vehicle to extend the residual period of a drug at the wound area. As a vehicle for delivery of DMOG to oral mucosa in this study, was used a hyaluronic acid hydrogel due to its high viscosity and excellent biocompatibility; less cytotoxic and no inflammatory or allergic effects. However, the oral environment is extremely dynamic. Food, saliva, and drinking water can quickly remove topically applied hydrogels. In the present study, the porous structure of the hyaluronic acid hydrogel was not observed on the H-E stained sections, indicating that hyaluronic acid and incorporated DMOG had been washed away from the wound area before sacrifice on day 7. Therefore, it was not possible to estimate a valid amount of DMOG for wound healing. In this study, DMOG was applied at 0.5 and 1 mg/ml. A modest but statistically significant reduction in the wound area and distance occurred only at 1 mg/ml (Figs. 4 and 5). A more durable vehicle and increased concentration of DMOG could further accelerate healing of the palatal wound. However, the results of this study did demonstrate that HIF-1α is a potent target of wound healing in oral tissues.

## Conclusions

The expression of VEGF mRNA in RP cells was enhanced by DMOG. VEGF and HIF-1α protein expression were also increased significantly by treatment with DMOG. Topical application of 1 mg/ml DMOG with hyaluronic acid ointment significantly accelerated healing of palatal wounds in rats. These results demonstrate the usefulness of DMOG for promotion of wound healing of oral soft tissues.

## Abbreviations

DMEM: Dulbecco’s modified Eagle’s medium
DMOG: Dimethyloxalylglycine
HA: Hyaluronic acid
HIF-1α: Hypoxia inducible factor-1 alpha
PHDs: Prolyl hydroxylases
RP: Rat palatal
TGF-β: Transforming growth factor β
VEGF: Vascular endothelial growth factor
